# Improvement of *Verticillium* Wilt Resistance by Applying Arbuscular Mycorrhizal Fungi to a Cotton Variety with High Symbiotic Efficiency under Field Conditions

**DOI:** 10.3390/ijms19010241

**Published:** 2018-01-13

**Authors:** Qiang Zhang, Xinpeng Gao, Yanyun Ren, Xinhua Ding, Jiajia Qiu, Ning Li, Fanchang Zeng, Zhaohui Chu

**Affiliations:** 1 State Key Laboratory of Crop Biology, College of Agronomy, Shandong Agricultural University, Tai’an 271018, China; zqsdau@163.com (Q.Z.); gaoxinpeng1990@foxmail.com (X.G.); xhding@sdau.edu.cn (X.D.); chunshuijiaren@163.com (J.Q.); nli@sdau.edu.cn (N.L.); 2 Shandong Provincial Key Laboratory of Vegetable Disease and Insect Pests, College of Plant Protection, Shandong Agricultural University, Tai’an 271018, China; 3 Jining Academy of Agricultural Sciences, Jining 272031, China; renyanyun@126.com

**Keywords:** mycorrhizal colonization, *Gossypium hirsutum*, *Verticillium* wilt, symbiotic efficiency, plant growth promotion, resistance, antifungal activity

## Abstract

Arbuscular mycorrhizal fungi (AMF) play an important role in nutrient cycling processes and plant stress resistance. To evaluate the effect of *Rhizophagus irregularis* CD1 on plant growth promotion (PGP) and *Verticillium* wilt disease, the symbiotic efficiency of AMF (SEA) was first investigated over a range of 3% to 94% in 17 cotton varieties. The high-SEA subgroup had significant PGP effects in a greenhouse. From these results, the highest-SEA variety of Lumian 1 was selected for a two-year field assay. Consistent with the performance from the greenhouse, the AMF-mediated PGP of Lumian 1 also produced significant results, including an increased plant height, stem diameter, number of petioles, and phosphorus content. Compared with the mock treatment, AMF colonization obviously inhibited the symptom development of *Verticillium dahliae* and more strongly elevated the expression of pathogenesis-related genes and lignin synthesis-related genes. These results suggest that AMF colonization could lead to the mycorrhiza-induced resistance (MIR) of Lumian 1 to *V. dahliae*. Interestingly, our results indicated that the AMF endosymbiont could directly inhibit the growth of phytopathogenic fungi including *V. dahliae* by releasing undefined volatiles. In summary, our results suggest that stronger effects of AMF application result from the high-SEA.

## 1. Introduction

Cotton (*Gossypium* spp.) is an essential resource for thousands of consumables and industrial products manufactured across the world. It continues to grow in importance in the fiber and oil industries. However, *Verticillium* wilt caused by the soil-borne fungus *Verticillium dahliae*, also known as the “cancer” of cotton crops, has become one of the most devastating diseases in cotton-growing areas, resulting in significant losses of plant biomass, lint yield, and fiber quality worldwide [1,2]. This pathogenic fungus can persist alone in the soil for up to 15 years by forming microsclerotia as resting structures [3]. Because of its highly variable pathogenicity and strong vitality, it is extremely difficult to control *Verticillium* wilt disease [4].

In China, approximately 2.5 million hectares of the cotton are affected by *Verticillium* wilt, which may cause direct economic loss of 250–310 million US dollars annually [4]. More seriously, in some regions, the losses of lint cotton yield may be as high as 80% [5]. Unfortunately, to date no effective fungicides are available to control this pathogen because of the strong viability of its microsclerotia along with its wide host range and highly variable pathogenicity [6]. Although many island cotton varieties (*Gossypium barbadense*) possess resistance to *Verticillium* wilt, upland cotton (*Gossypium hirsutum*), the commercial cultivar that accounts for more than 95% of the annual global cotton crop, lacks germplasm resources resistant to this disease [7]. Traditional cross-breeding between the two species has not been successful because of hybrid dysgenesis, linkage drag, or abnormal separations in the progeny [8,9].

Histochemical analyses have revealed that intrinsic defenses play a dominant role in the disease resistance of island cotton, while such induced resistance is severely limited in upland cotton [10]. During the past decades, soil solarization and fumigation have been used as primary management strategies to reduce microsclerotia. However, in addition to playing only a limited role, they pose costs to the economy, ecological balance, and public health [11]. Currently, the molecular mechanisms underlying cotton resistance to *Verticillium* wilt are still poorly understood [12,13]. With the rapid development of genetic engineering, several genes have been identified to improve the cotton *Verticillium* wilt resistance, such as *GAFP4* [4] and a series of *Ve1* gene homologs including *Gbvdr5* [14], *Gbvdr3* [15], *GbaVd1,* and *GbaVd2* [16]. Alternatively, transgenic cotton that produced the RNAi construct of *VdH1* (VdH1i)-derived siRNAs showed efficient protection against *V. dahliae* [17]. They are potential sources to introduce into integrated pest management in the future.

Many reports emphasize that an induced resistance mechanism using mycorrhiza-induced resistance (MIR) rather than increased tolerance or other effects plays a major role in plant defense against a broad spectrum of pathogens [18,19,20,21]. MIR, a mild but efficient activation of the plant immune response, not only locally but also systemically shares some characteristics with systemic acquired resistance (SAR) after pathogen invasion and induced systemic resistance (ISR) triggered by non-pathogenic rhizobacteria [22]. At the precontact phase of mycorrhizal symbiosis, plant roots release strigolactones (which may also include an *N*-acetylglucosamine-based molecule) to induce the germination of fungal spores and stimulate hyphal branching [23,24]. Subsequently, arbuscular mycorrhizal fungi (AMF) produce lipochitooligosaccharides and chitooligosaccharides, which can be recognized by the host roots and then activate the symbiosis signaling pathway [25]. During this period, the innate immune system of host plant recognizes the microbe-associated molecular patterns (MAMPs) from AMF and initiates the transient expression of MAMP-triggered immunity (PTI), as well as the accumulation of the plant defense hormone salicylic acid (SA) in the vascular tissues [26]. Although the initial SA accumulation is restrained during successive stages of AMF infection, the primed defense state of SA-dependent defense and SAR can be sustained for long periods [27,28]. To establish a successful infection, AMF secrete specific effectors to suppress PTI and transiently induce the production of abscisic acid (ABA) in the roots [29]. ABA can be transported through the xylem to the phloem, by which it primes cell wall defense [30]. With the development of symbiosis and the modulation of plant immunity, the root exudation chemistry changes and then results in the delivery of ISR-eliciting signals by the mycorrhizosphere bacteria, which can be perceived by the host plant. Subsequently, the host plant generates long-distance signals that prime jasmonate- and ethylene-dependent plant defenses and cause ISR [18,31]. In summary, MIR can induce a primed state of the mycorrhizal plants that allows a more effective activation of defense mechanisms to address environmental challenges [22].

AMF are generally endomycorrhizal fungi of the phylum *Glomeromycota*, which can establish symbionts with most types of terrestrial plants [32]. The growth and development of AMF rely on the colonization of host roots to obtain their sugar and lipids [33,34]. In return, the AMF benefit the host plants, such as by helping to absorb and deliver phosphorus, enhancing resistance to biotic and abiotic stresses, and stimulating growth promotion [35,36,37]. In the past, research on AMF primarily focused on its beneficial effects on plant growth and nutrition, while recent studies have paid more attention to its biocontrol potential, in particular for soil-borne pathogens. AMF have exhibited inhibitory effects on several soil-borne pathogens including species of *Rhizoctonia* [38], *Verticillium* [39], *Fusarium* [40], *Phytophthora* [41], *Macrophomina* [42], and *Aphanomyces* [43]. Considering its unique advantages in food security, environmental protection and low risk of antimicrobial resistance, AMF should provide new avenues to protect cotton from the fungal pathogen *V. dahliae* in sustainable and organic agriculture.

Although it has been confirmed that AMF can benefit cotton growth and may behave as a biocontrol agent against *Verticillium* wilt [39,44], studies of symbiotic efficiency between AMF and different cotton cultivars have not yet been reported, and the effects of plant growth promotion (PGP) or resistance towards *V. dahliae* by AMF on high-SEA cotton varieties have never been investigated. The main objective of this study was to assess the potential of AMF *Rhizophagus irregularis* CD1 in controlling *Verticillium* wilt in high-SEA cotton cultivars directly in the field.

## 2. Results

### 2.1. Symbiotic Efficiency of Seventeen Cotton Varieties Inoculated by Arbuscular Mycorrhizal Fungi (AMF) R. irregularis

**Figure 1:**
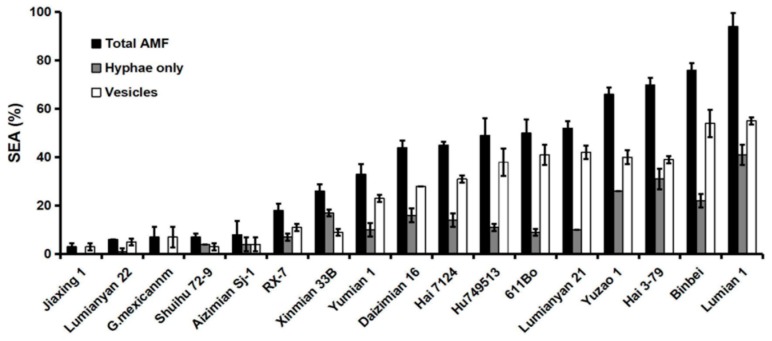
Evaluation of the symbiotic efficiency of AMF (SEA) among seventeen cotton cultivars. One hundred 1-cm root fragments were investigated from each plant after 40 days of growth with *Rhizophagus irregularis* CD1 during greenhouse conditions. The parameters Hyphae only (%), Vesicles (%), and Total AMF (%) denote the frequency of internal hyphae (without vesicles), vesicles, and total mycorrhizal colonization, respectively. The experiment was repeated twice under greenhouse conditions. Error bars represent ± SD.

Sufficient mycorrhizal colonization is one of the key factors to initiate the symbiotic system. To select an appropriate cotton variety for this study, we first investigated the SEA of 17 cotton varieties at 40 days post inoculation (dpi) under greenhouse conditions. A variety of infection structures could be observed in the roots, including vesicles, hyphae, spores and arbuscules (Figure S1). Based on two repeated experiments, we found that the total SEA ranged dramatically from 3% to 94% with the 17 cotton varieties tested. Lumian 1 is one of the highest SEA (SEA = 94%) varieties. In addition, the colonization ratio of hyphae and vesicles was inconsistent in different varieties, and Lumian 1 had the highest ratio, reaching 41% and 55%, respectively (Figure 1).

### 2.2. Effect of Plant Growth Promotion (PGP) among Cotton Varieties with Different Symbiotic Efficiency of AMF (SEA)

**Figure 2:**
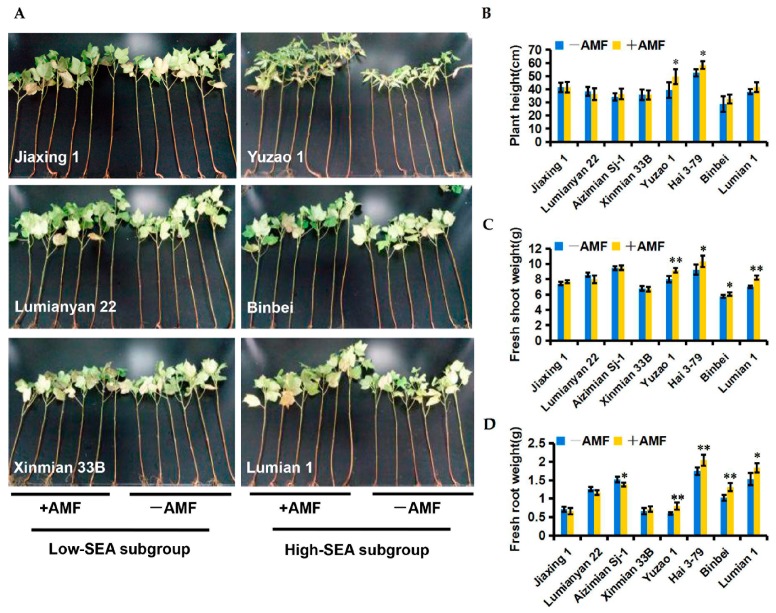
Effects of different symbiotic efficiency of AMF (SEA) on cotton growth promotion at 60 days post inoculation (dpi) under greenhouse conditions. +AMF: mycorrhizal; −AMF: nonmycorrhizal. (**A**) The growth phenotypes of two cotton subgroups. The low-SEA subgroup (SEA ≤ 26%) was comprised of Jiaxing 1, Lumianyan 22, Aizimian Sj-1, and Xinmian 33B. The high-SEA subgroup (SEA ≥ 66%) was comprised of Lumian 1, Binbei, Hai 3-79, and Yuzao 1; (**B**–**D**) Biomass statistics of two cotton subgroups including plant height (**B**), shoot fresh weight (**C**), and root fresh weight (**D**). Error bars represent ± SD. * *p* < 0.05; ** *p* < 0.01.

To test the effect of PGP among different SEA cotton varieties, two subgroups that contained four cotton varieties, each with high-SEA and low-SEA, were inoculated with *R. irregularis* CD1 or water (control) under greenhouse conditions, respectively. As expected, AMF-mediated PGP was dependent on the SEA. In the high-SEA subgroup (SEA ≥ 66%), AMF colonization resulted in a significant increase in plant growth parameters including shoot fresh weight and root fresh weight. In addition, treatment of Yuzao 1 and Hai 3-79 resulted in a significant level of promotion in plant height (Figure 2A–D). In contrast, the low-SEA subgroup (SEA ≤ 26%) did not show any positive growth responses in their plant height and shoot and root fresh weights (Figure 2A–D). This suggests that the beneficial effects of AMF on cottons depend on a highly defined symbiotic relationship.

### 2.3. Field Evaluation of PGP of Lumian 1 by Applying for AMF

Given the highest SEA of Lumian 1 and its effective growth promotion associated with AMF under greenhouse conditions, we expected that it could be extended into the field. Lumian 1 is one of the leading cultivars in China because of its high yield and outstanding quality. However, it is not resistant to *Verticillium* wilt [45]. Therefore, it is a good candidate to use to appraise the potential of AMF application to improve plant growth and *Verticillium* wilt resistance.

**Figure 3:**
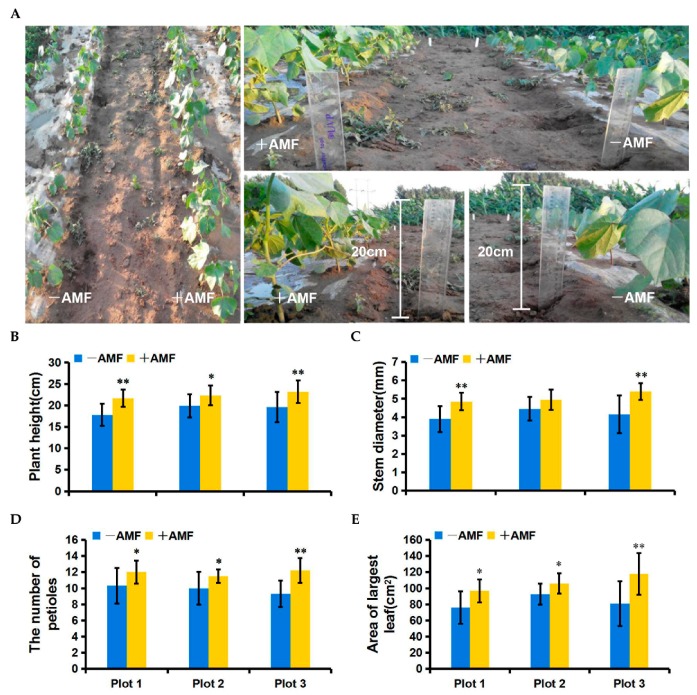
Effects of AMF on the growth of Lumian 1 at 40 dpi under field conditions. +AMF: mycorrhizal; −AMF: nonmycorrhizal. (**A**) The growth phenotypes of Lumian 1. (**B**–**E**) Biomass statistics of Lumian 1 including plant height (**B**), stem diameter (**C**), the number of petioles (**D**), and the area of the largest true leaf (**E**). Error bars represent ± SD. * *p* < 0.05; ** *p* < 0.01.

**Figure 4:**
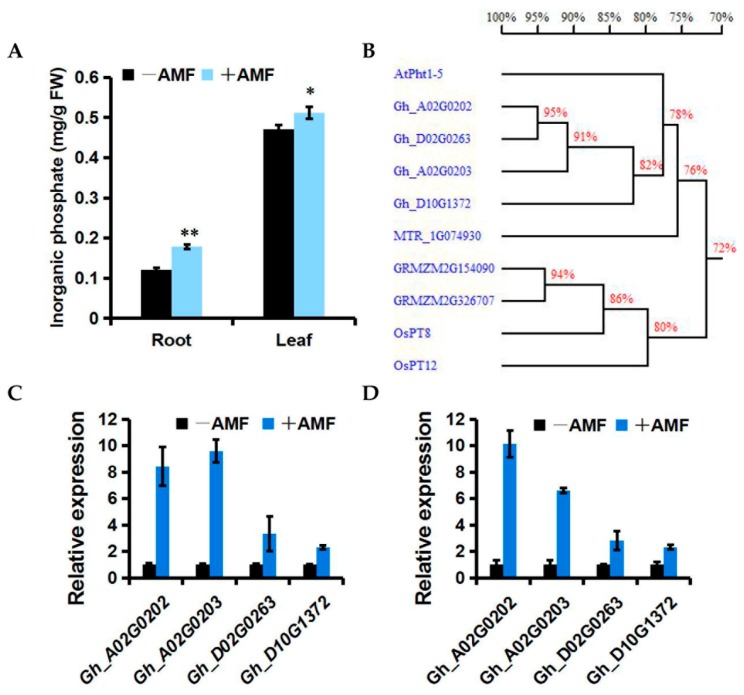
Effects of AMF on inorganic phosphorus (Pi) transport of Lumian 1 at 60 dpi under field conditions. (**A**) Pi content of both the root and leaf. +AMF: mycorrhizal; −AMF: nonmycorrhizal. Error bars represent ±SD. * *p* < 0.05; ** *p* < 0.01. (**B**) Homology tree for amino acid sequences of AtPht 1–5 (At2G32830) and its homologs within *Gossypium hirsutum* (Gh_A02G0202, Gh_A02G0203, Gh_D02G0263 and Gh_D10G1372), *Oryza sativa* (OsPT8, AAN39049; OsPT12, AAN39053), *Zea mays* (GRMZM2G326707, GRMZM2G154090) and *Medicago truncatula* (MTR_1g074930). This was generated by DNAMAN version 5.2.2.0 (Lynnon Biosoft, San Ramon, CA, USA); (**C**,**D**) Expression level of cotton homologs of *AtPht 1–5* in root (**C**) and leaf (**D**). The test performed by quantitative RT-PCR analysis. Transcript abundance of genes was normalized to that of the reference gene *UBQ7* (GenBank Accession Number: DQ116441). Three biological replicates were used for each reaction with three technical replicates each. Mean values and standard errors were calculated from three biological replicates.

We first conducted a field trial for AMF-mediated PGP in 2015. At 40 dpi, mycorrhiza-treated cotton grew significantly better than the control (Figure 3A). Statistical analyses revealed that the AMF applications significantly enhanced almost all of the growth parameters including plant height, stem diameter, number of petioles, and the area of the largest functional leaf (Figure 3B–E). Obvious effects of PGP could sequentially be observed by applying the AMF at 55 dpi and 72 dpi in both 2015 and 2016 (Table S1). Compared with the control, the mycorrhiza-treated cotton exhibited a higher inorganic phosphorus (Pi) content in both the roots and leaves (Figure 4A). Correspondingly, the transcription levels of several phosphate transport genes, including Gh_A02G0202, Gh_A02G0203, Gh_D02G0263, and Gh_D10G1372, which shared 78%, 77%, 77%, and 78% similarity with the phosphate transporter 1–5 gene (At2G32830) from *Arabidopsis thaliana*, respectively, were significantly induced in both the roots and leaves by AMF colonization (Figure 4B–D). This implies that these genes may contribute to the Pi transport from AMF to cotton during mycorrhizal symbiosis. Consistent with the benefits from AMF in a greenhouse assay, AMF is also highly effective at improving the plant growth of high SEA cotton varieties under field conditions.

### 2.4. Field Performance of Lumian 1 Inoculated with AMF for Verticillium Wilt Resistance

**Figure 5:**
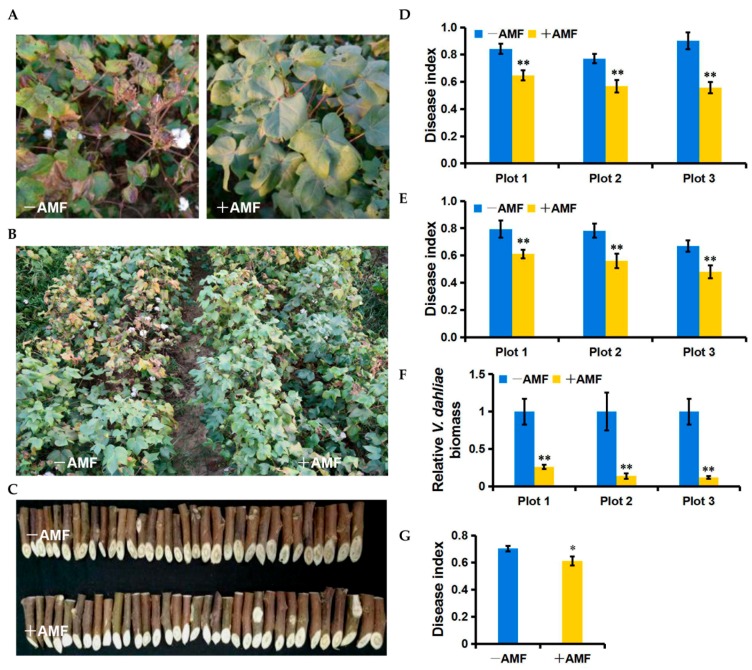
Effects of AMF on the *Verticillium* wilt resistance of Lumian 1 under field conditions. +AMF: mycorrhizal; −AMF: nonmycorrhizal. (**A**) The phenotypes of Lumian 1 at the early occurrence phase of *Verticillium* wilt; (**B**) The resistance phenotypes of Lumian 1 at 120 dpi in 2016; (**C**) The vascular discoloration phenotypes of Lumian 1 at 150 dpi in 2016; (**D**,**E**) The disease index of leaves at 120 dpi in 2015 (**D**) and 2016 (**E**), respectively. At least 40 plants were used for each experiment; (**F**) Quantitative detection of the *V. dahliae* biomass relative to cotton leaves at 120 dpi in 2016. The average fungal biomass was determined using at least 10 mycorrhiza-treated and 10 control cottons of each plot; (**G**) The disease index of the vascular bundle at 150 dpi in 2016. Error bars represent ± SD. * *p* < 0.05; ** *p* < 0.01.

Over two years of independent field trials, we discovered that the application of AMF delayed the symptoms of *Verticillium* wilt disease. With the early outbreak of the disease, mycorrhizal plots exhibited fewer chlorotic and necrotic spots than the control, and typically, this was the only place that healthy plants were visible (Figure 5A); conversely, the control plots commonly displayed susceptibility in their leaves, and some plants eventually died (Figure 5B). These results indicated that AMF colonization is likely to effectively inhibit the spread of *V. dahliae* in cotton at its early occurrence phase. In 2015, the disease index (DI) of three mycorrhiza-treated plots showed reductions of 23.10%, 26.36%, and 38.18% compared to each control plot, respectively (Figure 5D). In 2016, the DI decreased by 22.98%, 28.29%, and 28.41% in three mycorrhiza-treated plots compared to the control, respectively (Figure 5E). Interestingly, we noticed three disease centers that were distributed among the three control plots in the field (Figure S2B). Consistent with the reduction of DI by applying the AMF, the *V. dahliae* biomass relative to the cotton leaves was significantly lower in the mycorrhiza-treated plots than in the control plots (Figure 5F). In addition to changes in symptoms in the leaves, we found less vascular discoloration in the mycorrhiza-treated plots than in the control plots (Figure 5C). Statistical analyses revealed that the AMF treatment significantly reduced vascular discoloration by 13.12% (*p* = 0.05) (Figure 5G). Therefore, we concluded that AMF treatment can efficiently enhance the resistance of Lumian 1 to *Verticillium* wilt under field conditions.

### 2.5. Effect of Mycorrhizal Colonization on Cotton Resistance-Related Genes

**Figure 6:**
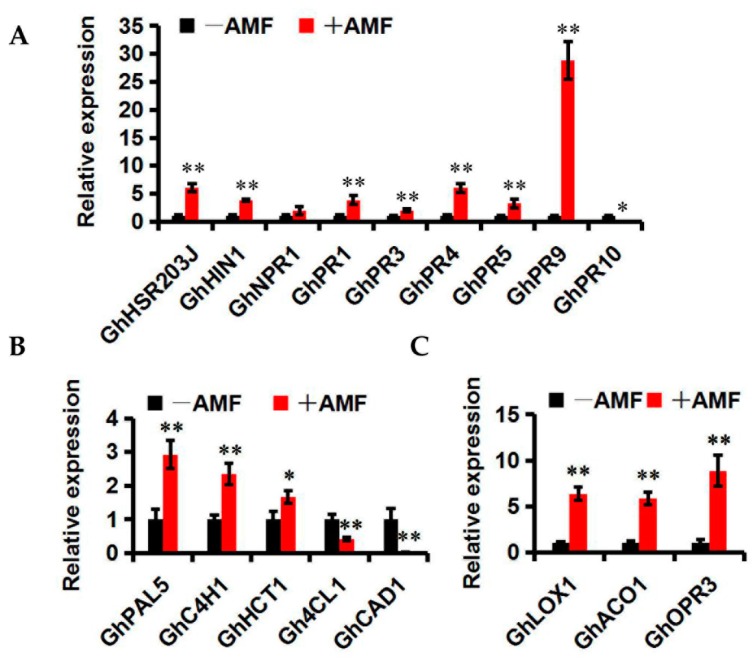
Expression patterns of cotton resistance-related genes in mycorrhizal (+AMF) and nonmycorrhizal (−AMF) Lumian 1. (**A**) Expression of *PR* genes; (**B**) Expression of JA synthesis-related genes; (**C**) Expression of lignin synthesis-related genes. The test was performed using reverse transcription quantitative PCR analysis of relative gene expression. Transcript abundance of genes was normalized to that of the reference gene *UBQ7* (GenBank Accession Number: DQ116441). Three biological replicates were used for each reaction with three technical replicates each. Mean values and standard errors were calculated from three biological replicates. * *p* < 0.05; ** *p* < 0.01.

SAR is a common resistance reaction directed against many types of phytopathogens. Some biochemical changes appear in the plant cells during SAR, such as the biosynthesis of pathogenesis-related (PR) proteins that are induced by stresses and play important roles in plant defense. *GhHSR203J* and *GhHIN1*, which are considered as marker genes for the hypersensitive response [46,47], were up-regulated by mycorrhizal colonization compared with the control (Figure 6A). *GhPR1* is a biomarker gene of SA signaling pathways [48]. *GhPR3* and *GhPR4* degrade chitin. *GhPR5* is thought to be involved in the synthesis of enzymes that metabolize and inhibit plant pathogens. *GhPR9* is a peroxidase that can reinforce the plant cell wall by catalyzing the synthesis of lignin to defend against the pathogen infection [48]. *GhPR1*, *GhPR3*, *GhPR4*, *GhPR5*, *and GhPR9* also exhibited increases of 3.8-, 2.0-, 2.0-, 3.3-and 28.8-fold in mycorrhiza-treated cotton plants, respectively, compared to the controls (Figure 6A). *GhPR10*, which may play a negative regulation role in SAR [49], was significantly reduced to 0.04-fold by applying AMF. These data suggest that the intensity of SAR can be further enhanced by mycorrhizal colonization.

Lignin synthesis is important in the resistance of cotton to *V. dahliae* [50,51]. To investigate its function during mycorrhizal colonization, the expression of lignin synthesis-related genes was examined, including *GhHCT1*, *GhPAL5*, *GhC4H1*, *Gh4CL1*, and *GhCAD1*. Compared with the controls, higher transcription levels of *GhHCT1*, *GhPAL5*, and *GhC4H1* and lower transcription levels of *Gh4CL1* and *GhCAD1* were detected in mycorrhiza-treated cotton (Figure 6B), indicating that AMF are capable of resisting *V. dahliae* by mediating lignin synthesis.

Jasmonic acid (JA) can accumulate during mycorrhizal symbiosis and contribute to MIR [22,52]. The expression of three biomarker genes of JA signaling pathways including *GhLOX1*, *GhACO1* and *GhOPR3* [53] was significantly up-regulated by 6.34-, 5.87- and 8.87-fold, respectively, after mycorrhizal colonization (Figure 6C), indicating that JA biosynthesis may be influenced by AMF colonization.

### 2.6. In Vitro Antifungal Activity Assay of AMF Symbionts

**Figure 7:**
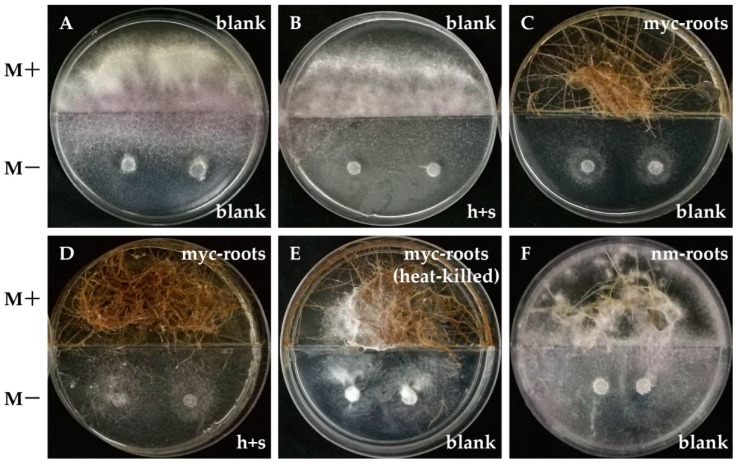
In vitro antimicrobial activities of AMF against *Verticillium dahliae*. Divided Petri dishes prevent nonvolatile solutes from diffusing between two compartments. The upper compartment of divided Petri dishes contained a complete growth medium (M+) used for the growth of AMF symbionts, and the lower compartment contained the same medium lacking sugar (M−), thus permitting the development of AMF hyphae and spores. Two discs covered with *V. dahliae* of 5 mm diameter were transferred to the M− compartment and incubated in the dark at 25 °C for 1 week. Before the inoculation of *V. dahliae* discs, fresh blank M+ and blank M− medium were decanted into divided Petri dishes. The height of M− was equal to the middle baffle while slightly higher than M+. The heights of M+ and M− in each plate were comparable to each other. Treatments were divided as follows: (**A**) empty M− and empty M+; (**B**) M− with AMF hyphae and spores (h + s) and empty M+; (**C**) empty M− and M+ with mycorrhizal roots (myc-roots); (**D**) M− with AMF h + s and M+ with myc-roots; (**E**) empty M− and M+ with dead myc-roots heated at 65 °C for 30 min; (**F**) empty M− and M+ with non-mycorrhizal roots (nm-roots). Experiments were repeated three times with similar results.

**Figure 8:**
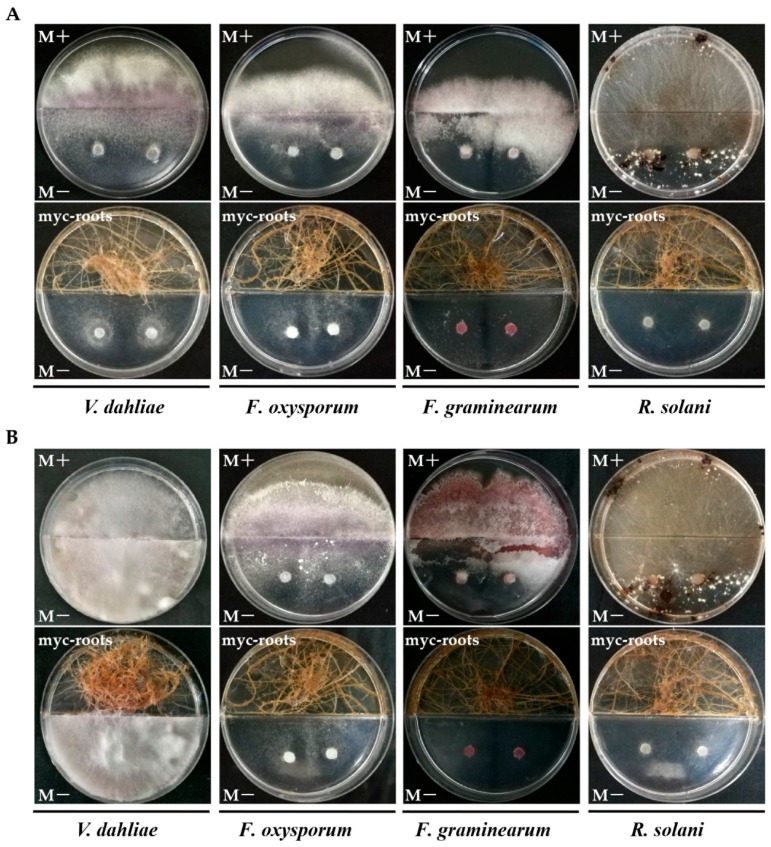
Antifungal activities of AMF symbionts against several soil-borne fungi at 7 dpi (**A**) and 50 dpi (**B**), respectively. The culture conditions were as described for Figure 7. The upper compartment contained M+ medium with or without AMF symbionts (myc-roots), and the downward compartment contained M− medium inoculated with two discs of *Verticillium dahliae*, *Fusarium oxysporum*, *Fusarium graminearum* and *Rhizoctonia solani*. Experiments were repeated three times with similar results.

The ability of AMF hyphae, spores and endosymbionts to inhibit the growth of *V. dahliae* in in vitro systems was tested first. Surprisingly, we found the AMF symbionts, rather than AMF hyphae or spores, caused an obvious inhibitory effect on the mycelial growth of *V. dahliae* (Figure 7). As shown in Figure 7, the mycelia of *V. dahliae* could quickly spread to the M+ compartment when the mycorrhizal roots were absent (Figure 7A,B,E,F), but their growth was limited in the M− compartment (M medium without sugar) when the mycorrhizal roots were planted in the M+ compartment (Figure 7C,D). Intriguingly, we subsequently demonstrated that AMF symbionts could also remarkably inhibit the growth of *Fusarium oxysporum*, *Fusarium graminearum*, and *Rhizoctonia solani* at 7 dpi (Figure 8A). Even at 50 dpi, there was almost no mycelial expansion of *F. graminearum* and no sclerotial production of *R. solani* when AMF symbionts were present (Figure 8B). Therefore, it seems as if mycorrhizal colonization can not only enhance the resistance of host plants to *V. dahliae* via inducing the expression of resistance-related genes but may also have a direct adverse effect on fungi by its symbionts which may generate certain volatile compounds.

## 3. Discussion

Numerous studies have shown that AMF can contribute to the growth and disease resistance of host plants. Our study presents interesting results about the effects of symbiotic efficiency of AMF (SEA) on the cotton growth improvement. We corroborated that high-SEA cotton Lumian 1 could perform well on both growth promotion and *Verticillium* wilt resistance following mycorrhizal colonization directly under field conditions. In addition to its roles in inducing plant disease resistance, we revealed that AMF symbionts could release certain volatile compounds, which may have broad-spectrum and long-term efficacy in terms of fungistasis.

AMF are the most widespread endomycorrhizal fungi, which play important roles in nutrient cycling processes and plant stress resistance by establishing symbiotic associations with plant roots. AMF can increase plant nutrient uptake of P [54,55], N [56,57], and K [58]. In addition, AMF are beneficial to the stabilization of soil aggregates [59], and improve resistance to water stress [36] and defense against pathogens [18]. However, a successful symbiotic relationship between AMF and its host involves the premise that the AMF will have an effect on improving plant growth and disease tolerance. The viability of AMF application in agricultural soils depends on many factors, including species compatibility, habitat niche availability, and competition with indigenous fungi [60]. To date, significant genetic variation of AMF species in their effects on host plants has been reported [61,62,63]. However, few studies have focused on the impact of diverse genotypes on both the plant response to the AMF as well as its mycorrhizal colonization level. Cotton is a mycotrophic plant in which growth and nutrient uptake is usually promoted by mycorrhizal colonization [64]. Here, we used SEA to appraise the level of mycorrhizal colonization in 17 cotton varieties under greenhouse conditions. Remarkably, the total SEA varied widely ranged from 3% (Jiaxing 1) to 94% (Lumian 1) among the cotton varieties tested (Figure 1). Eaton et al. inoculated 43 near-isogenic lines of *Trifolium repens* with AMF *Glomus mosseae* and observed a high degree of variation among individual lines in their mycorrhizal root infection rates [65]. Therefore, although AMF can colonize the roots of more than 90% of plant species, to a typical species, the ratio of mycorrhizal colonization is subject to the genotypes that imply its control by genetic characteristics.

We speculate that an effective SEA may be elementary for host plants to benefit from mycorrhizal colonization. Consistent with this hypothesis, AMF significantly improved plant growth of the high-SEA subgroup but did not do so on the low-SEA subgroup in the greenhouse (Figure 2A–D). Consistent with a role as a potential biofertilizer, the high performance of AMF-mediated PGP with Lumian 1 was also observed under field conditions (Figure 3A–E). This could be partially explained by helping the host plant to take up components such as phosphorus (P), which is a well-known contribution from mycorrhizal symbiosis [66]. Our results showed that mycorrhizal colonization significantly enhanced the inorganic phosphate (Pi) content in both the roots and leaves relative to the control (Figure 4A). Since *Pht 1–5* plays vital roles in Pi translocation and remobilization [67], its co-expression with the accumulation of Pi, the expression of certain cotton homologs of *AtPht 1–5*, including *Gh_A02G0202*, *Gh_A02G0203*, *Gh_D02G0263* and *Gh_D10G1372*, were induced in all of the mycorrhiza-treated plots (Figure 4B–D). In combination, an effective SEA is required for AMF-mediated PGP, including but not limited to providing predominantly Pi in exchange for the plant carbon source. However, SEA is highly variable in its host genotype. This genotypic variation is worth harnessing in genetic breeding to fully exploit the potentials of AMF, especially for regions of the world with Pi deficiency.

The other benefits of symbiotic systems include the promotion of the host resistance. In two-year field trials to control *Verticillium* wilt, we demonstrated a significant reduction of disease severity by AMF application with Lumian 1 (Figure 5A–G). Generally, the mechanisms of plant resistance can be divided into two broad categories: constitutive resistance and induced resistance. Lignin synthesis, a major constitutive resistance mechanism that involves the formation of new cell walls as a response to pathogens, has been shown to play a central role in the resistance of cotton to *V. dahliae*. The content of lignin in island cotton is higher than that in upland cotton, which is one of the core reasons that the former is more resistant to *V. dahliae* [50,51]. A reduction in the lignin content by silencing *GhHCT1* with VIGS in cotton had resulted in compromised resistance to *V. dahliae* [51]. This was accompanied by the reduced transcription level of upstream lignin synthesis-related genes such as *GhPAL5* and *GhC4H1* and the increased expression of genes involved in downstream lignin synthesis including *Gh4CL1* and *GhCAD1* [51]. Consistent with the higher level of resistance to *Verticillium* wilt, higher transcription levels of *GhHCT1*, *GhPAL5*, and *GhC4H1* and lower transcription levels of *Gh4CL1* and *GhCAD1* were detected in mycorrhiza-treated cotton, as compared to the control (Figure 6B), indicating that the AMF can resist *V. dahliae* by mediating lignin synthesis. MIR can be considered to be the other key reason to promote host resistance. The expression of many *PR* genes, including *GhHSR203J*, *GhHIN1*, *GhPR1*, *GhPR3*, *GhPR4*, *GhPR5*, and *GhPR9*, was significantly increased by mycorrhizal colonization. However, the expression of *GhPR10* was significantly reduced to 0.04-fold after AMF application (Figure 6A). Some PR10 homologs were shown to display antifungal activities in different plant species [68,69]. In contrast, the overexpression of *STH-2*, a member of the *PR10* family, in potato failed to enhance the resistance of potato to *Phytophthora infestans* and potato virus X [70]. Similar results were also identified in other studies [49,71]. In cotton, it appeared that a high resistance to *V. dahliae*, as well as more lignin, accumulated in the stems when *GhPR10* was silenced by VIGS, suggesting that *GhPR10* probably negatively regulates the resistance of cotton to *V. dahliae* [72,73]. AMF can affect the transcription of the *PR* genes and further enhance the intensity of SAR. In addition, the up-regulation of *GhLOX1*, *GhACO1* and *GhOPR3*, which encode key enzymes involved in JA biosynthesis pathway [53], was observed in mycorrhiza-treated cotton (Figure 6C), consistent with the concept that JA can accumulate during mycorrhizal symbiosis and contribute to MIR [22,52]. We conclude that the significant resistance to *V. dahliae* resulting from mycorrhizal colonization in high-SEA cotton Lumian 1 is associated with MIR and cell wall secondary metabolism.

In addition to MIR and cell wall defense, an antifungal activity of root exudates induced by AMF symbiosis was observed to promote resistance. *Glomus versiforme* could alter the exudation pattern of cotton roots and contribute to the bioactive effects on *V. dahliae* conidial germination [74]. *Pisolithus tinctorius* strain SMF, an ectomycorrhizal fungus, strongly inhibited the growth of *V. dahliae* [75]. Ericoid mycorrhizal fungi could protect the host plants from infections by pathogens such as *Phytophthora cinnamomi* and *Pythium* when they were sufficiently present in or on the roots [76]. Hage-Ahmed et al. demonstrated that a direct antibiotic activity of root exudates from tomatoes towards *F. oxysporum* f.sp. *lycopersici* could be induced by the interactions of the plant–AMF–pathogen [77]. Using HPLC-UV analyses, the antifungal substances were identified to be nonvolatile citrate and chlorogenic acid [77]. Intriguingly, our results showed that the mycorrhizal roots of carrots, rather than AMF hyphae, AMF spores, or non-mycorrhizal roots, suppressed the mycelial growth of *V. dahliae* (Figure 7). Carrot roots also strongly inhibit the growth of other soil-borne fungi such as *F. oxysporum*, *F. graminearum* and *R. solani*. In particular, this fungistasis could persist as long as 50 dpi, while in the Petri dish, the mycelia of *F. graminearum* could rarely expand, in addition to the effects on *R. solani* that included a lack of sclerotia production (Figure 8A,B). Given the blocking effect of the divided Petri dishes on the diffusion of nonvolatile solutes from root exudates, we propose that AMF symbionts can release certain volatile compounds that directly inhibit the growth and extension of fungal phytopathogens, which is to the best of our knowledge a novel biocontrol mechanism of mycorrhizal association. However, there are still many questions that need to be investigated further, such as identifying the volatile compounds, and whether they are generated in the *R. irregularis* CD1-Lumain 1 interaction system, and whether they are involved in promoting the resistance to *Verticillium* wilt.

## 4. Materials and Methods

### 4.1. AMF Inoculum Preparation

*R. irregularis* CD1 was used in this study and was maintained on Petri dishes as described by St-Arnaud [78]. Briefly, *R. irregularis* CD1 was co-cultivated with genetically transformed carrot roots in a two-compartment in vitro system. Half Petri dishes containing a complete growth medium (M+) were used for the growth of endosymbionts, while the other half containing the same medium excluding sugar (M−), thus permitting the development of AMF mycelia and spores. The height of M− was equal to the middle baffle, while M+ was slightly lower than that. The Petri dishes were incubated in the dark at 18 °C. After 2–3 months, a greater number of spores could be easily seen on the M− compartment. The medium containing spores was then blended with distilled water in a juice blender. The mixture of mycelia and spores was agitated by a magnetic stirrer, and then the spores were counted using a hemocytometer and stored temporarily in a 4 °C refrigerator.

### 4.2. Evaluation of SEA among 17 Cotton Cultivars

The seeds of 17 cotton cultivars (Figure 1) were provided by the collections from the Cotton Research Institute, Chinese Academy of Agricultural Sciences. All seeds were villus-shed and surface-sterilized by using 98% H_2_SO_4_ (100 °C, 1 min) and then soaked in water at 28 °C until they germinated.

Barren soil was collected at the fields of Shandong Agricultural University (Taian, China). The germinated seeds were planted in 15-cm diameter plastic pots (4 L) containing the soil-vermiculite-perlite mixture (3:2:1 ratio, *v*/*v*) sterilized by steaming (121 °C for 30 min). To ensure the direct contact of the seedling roots with *R. irregularis* CD1, we added a suspension of approximately 2000 spores into the center of each pot, 3 cm under the surface of the mixture. Each pot was planted with five seeds of the same cotton variety, and three seedlings of similar development were reserved for further study. The experiment was carried out in a greenhouse at 60% relative humidity under a 16/8 h and 26/20 °C (light/dark) photoperiod. Plants were harvested, and roots from each pot were collected separately at 40 days post-inoculation (dpi). The experiment was repeated twice. Each pot was repeated three times.

To assess the mycorrhizal colonization of each cotton variety by *R. irregularis* CD1, we used Trypan blue staining as described by Phillips [79]. Briefly, root samples were thoroughly washed and cut into approximately 1 cm sections, then steeped in 10% KOH (*w*/*v*) for 45 min at 60 °C and stained with 0.05% *v*/*v* Trypan blue in lactic acid. Separately, 100 1-cm-long fine roots were randomly selected from each line. Symbiotic efficiency was assessed using a Nikon Eclipse 90i light microscope. An estimate of Total AMF (%) was given as the ratio between root fragments colonized by an AMF structure. The parameter Vesicles (%) was an estimate of vesicle richness in the whole analyzed root system. The parameter Hyphae only (%) was the proportion of the root cortex infected by hyphae that lacked vesicles relative to the whole root system analyzed.

### 4.3. Experimental Design for Testing the PGP and Wilt Disease Resistance

A pot experiment to test the PGP with eight cotton varieties was described as above. The matched controls were given the equivalent volume of blank M− suspension. Four high-SEA subgroups (SEA ≥ 66%) were selected as Lumian 1, Binbei, Hai 3-79 and Yuzao 1. The other four low-SEA subgroups (SEA ≤ 26%) included Jiaxing 1, Lumianyan 22, Aizimian Sj-1 and Xinmian 33B. At 60 dpi, plant height and shoot and root fresh weights of the whole plants were measured. This experiment was repeated three times. Each pot planted three cottons and was repeated twice.

Field performance was investigated twice in the plain area of Taian city during 2015 and 2016 where *Verticillium* wilt occurred. The cotton variety Lumian 1 of the highest SEA was selected in this experiment. The experimental design was a complete randomized block (three mycorrhiza-treated plots and three control plots). Each plot was 8 m in length and 1 m in width (with a 1 m interval between blocks), and 50 plants were planted in two rows in each plot. Plot 1, Plot 2 and Plot 3 were used to refer to three repetitions (Figure S2A). We inoculated each germinated seed with a suspension of 800 spores or an equal volume of blank M− suspension. At 40, 55 and 72 dpi, the growth parameters were recorded for ten randomly selected plants from each experimental plot. At 60 dpi, six plants were randomly collected from mycorrhiza-treated and control plots respectively to measure the Pi content and the expression of several phosphate transport genes in both the root and leaves.

Subsequently, we evaluated the biological control of *Verticillium* wilt using AMF application during two years of field trials. Disease severity was recorded for each plant at 120 dpi using a disease index (DI) ranging from 0 to 4 rating scale based on to the percentage of foliage affected by acropetal chlorosis, necrosis, wilt, and/or defoliation as follows: 0 = no visible disease symptoms, 1 = 1–25%, 2 = 26–50%, 3 = 51–75%, and 4 = 76–100% or a dead plant. We randomly gathered a mixture of leaf and root samples from ten mycorrhiza-treated and control plants in each plot, which were used to extract DNA to measure the *V. dahliae* biomass and RNA to detect the expression of cotton defense-related genes, respectively. To better assess the severity of the disease, we also estimated the vascular discoloration of the cotton stems at 150 dpi. Twelve stems were randomly collected from each mycorrhiza-treated and control plot to be rated for vascular discoloration. We established a new rating scale according to the proportion of stem length that occurred vascular browning in longitudinal section as follows: 0, no vascular discoloration, 1 = 1–25%, 2 = 26–50%, 3 = 51–75%, 4 = 76–100%.

### 4.4. RNA Extraction and Reverse Transcription Quantitative PCR Analysis

Total RNA was isolated from 50 mg plant tissue by a E.Z.N.A.™ Plant RNA Kit (OMEGA-biotek, R6827-01, Doraville, GA, USA). One-half microgram RNA was used for first-strand cDNA synthesis using the *EasyScript* ^®^One-Step gDNA Removal and cDNA Synthesis SuperMix (TRANs, Beijing, China). Quantitative PCR was performed with UltraSYBR Mixture (Comwin Biotech Co., Ltd., Beijing, China) on a QuantStudio™ 6 Flex Real-Time PCR System (Thermo Fisher, Waltham, MA, USA). The PCR program was as follows: 95 °C for 10 min, followed by 40 cycles of 95 °C for 15 s, 60 °C for 1 min. The specificity of the amplified PCR products was determined by melting curve analysis (95 °C for 15 s, 60 °C for 1 min, 95 °C for 15 s and 60 °C for 15 s). *UBQ7* of *G. hirsutum* (GenBank Accession Number: DQ116441) was used as internal control to standardize the results [80]. For each gene, quantitative PCR assays were repeated at least twice with triplicate runs. Relative expression levels were measured using the 2^−^^∆∆*C*t^ analysis method. The sequences of gene-specific primers used in the assay are listed in Table S2.

### 4.5. Quantitative Detection of V. dahliae Biomass in Cotton

At 120 dpi, we randomly gathered the first true leaves of plants from six plots and extracted DNA by CTAB method [81]. To detect the *V. dahliae* biomass, quantitative PCR was performed with the same agents and amplification conditions as described above. The internal transcribed spacer region of the ribosomal DNA was targeted to generate a 200 bp amplicon using the fungus-specific primer ITS1-F, and the *V. dahliae*-specific reverse primer ST-VE1-R [82]. *UBQ7* was used for equilibration of the different DNA samples. The average fungal biomass was determined using ten mycorrhiza-treated and ten control cotton plants for each plot. This experiment was repeated three times.

### 4.6. Antimicrobial Activities of AMF against Phytopathogenic Fungi In Vitro Systems

To test whether AMF could contribute antifungal activity, the symbiotic carrot roots were cultured as described above. Phytopathogenic fungi were preincubated on PDA medium at 25 °C for four days. On the M− compartments, we inoculated two 5-mm diameter discs of cultures of *V. dahliae*, *F. oxysporum*, *F. graminearum* and *R. solani*, respectively. The Petri dishes were incubated in the dark at 25 °C and then observed at 7 or 50 dpi. This experiment was repeated three times.

### 4.7. Measurement of Pi Content

Approximately 0.5 g frozen samples were assayed using an improved method described by Nanamori et al. [83]. Briefly, the frozen sample was ground with liquid nitrogen and homogenized in 1 mL 10% (*w*/*v*) perchloric acid (PCA). The homogenate was diluted 10 times with 5% (*w*/*v*) PCA and then incubated on ice for 30 min. After centrifugation at 10,000× *g* for 10 min at 4 °C, 500 μL supernatant was transferred and blended with 4.5 mL H_2_O and 5 mL chromogenic agent. This mixture was incubated in a water bath at 45 °C for 25 min. After being cooled at 4 °C, the absorbance was measured at 820-nm wavelength. The Pi content was calculated using the normalization of fresh weight. The chromogenic agent contents were as follows: ddH_2_O:3 M H_2_SO_4_:2.5% (*w*/*v*) Hexaammonium heptamolybdate tetrahydrate (Sinopharm Chemical Reagent Co., Ltd., Shanghai, China):5% (*w*/*v*) l-Ascorbic acid (Sinopharm Chemical Reagent Co., Ltd.) = 2:1:1:1 (volume ratio).

### 4.8. Data Analysis

The software DPS (Data Processing System, version 7.05, Hangzhou, China) was used to perform the statistical analyses. All data were subjected to analysis of variance by one-way analysis of variance (ANOVA). A post hoc analysis test LSD (least significant difference) was implemented to examine the significance of different treatment means against a standard control (*p* = 0.05 or 0.01).

## 5. Conclusions

In this study, we introduced the concept of SEA and ascertained a broad phenotypic variation of SEA between the interaction of *R. irregularis* CD1 and seventeen cotton cultivars. Intriguingly, we demonstrated that high-SEA is positively associated with the AMF-mediated PGP effects. In this study, we identified the cotton variety Lumian 1, which possessed the highest SEA and could perform well on both growth promotion and disease resistance following mycorrhizal colonization. These results suggest high-SEA is a key phenotype to accelerate the utilization of AMF that will contribute to research on the genetic improvement of SEA. The genotypic variation conferring high-SEA in Lumian 1 merits additional research to characterize this variation. In addition, we confirmed that the mycorrhizal roots of carrots could release unknown volatile compounds to directly inhibit the growth of several fungal phytopathogens, which to our knowledge, is a novel AMF-mediated biocontrol mechanism. The specific antifungal substances derived from the mycorrhizal symbionts merit additional research to identify these compounds.

## Supplementary Materials

Supplementary materials can be found at www.mdpi.com/1422-0067/19/1/241/s1.

## Abbreviations

AMF	Arbuscular mycorrhizal fungi
PGP	Plant growth promotion
SEA	Symbiotic efficiency of AMF
MIR	Mycorrhiza-induced resistance
SAR	Systemic acquired resistance
ISR	Induced systemic resistance
MAMP	Microbe-associated molecular patterns
PTI	MAMP-triggered immunity
SA	Salicylic acid
ABA	Abscisic acid
DI	Disease index
dpi	Days post inoculation
JA	Jasmonic acid
*PR*	Pathogenesis-related proteins
