# Supplementary material for: Improvement of Verticillium Wilt Resistance by Applying Arbuscular Mycorrhizal Fungi to a Cotton Variety with High Symbiotic Efficiency under Field Conditions
Source: Int J Mol Sci. 2018 Jan 13;19(1):241. doi: 10.3390/ijms19010241 (PMC5796189; doi:10.3390/ijms19010241)
Supplement: Supplementary file 1 [file ijms-19-00241-s001.pdf]

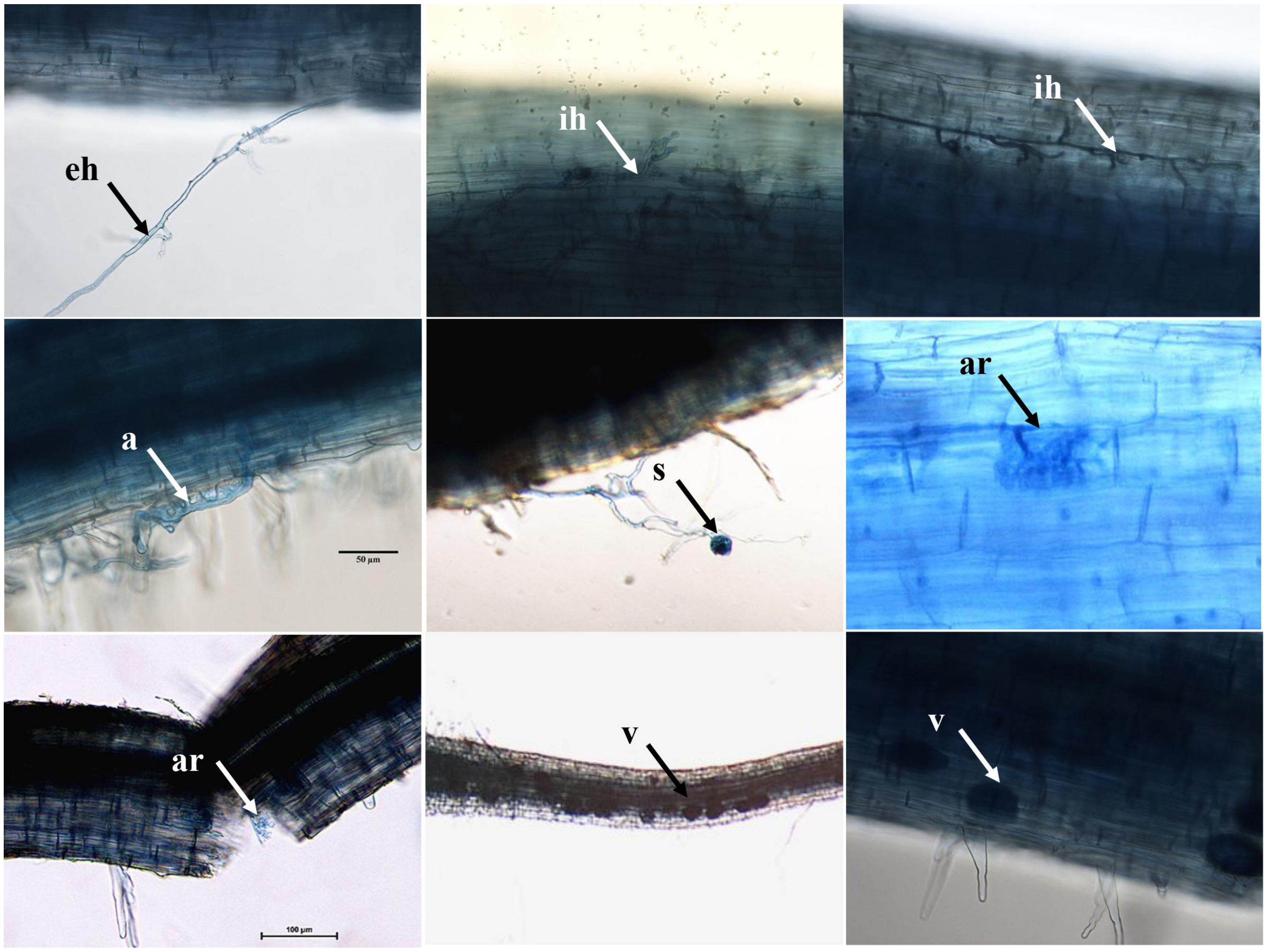

Figure S1. The morphology of cotton roots associated with arbuscular mycorrhizal fungi at 40 dpi. eh, extraradical hyphae; ih, intraradical hyphae; a, appressorium; s, spore; ar, arbuscules; v, vesicle.

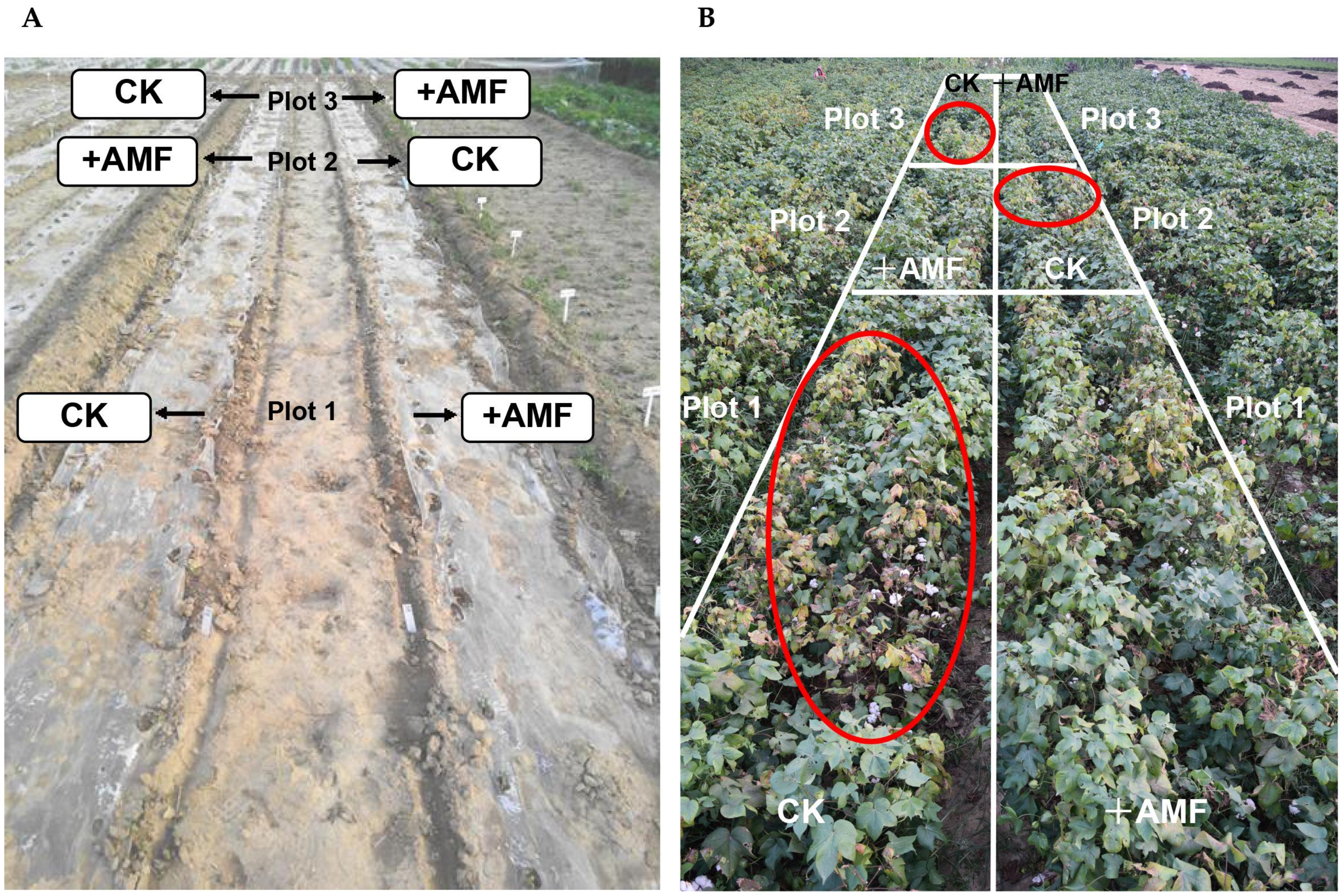

Figure S2. Experimental design and cotton wilt disease centers in the field in 2016. (A) Experimental design to test the PGP and Verticillium wilt resistance by applying AMF. The experimental design was a complete randomized block (three mycorrhiza-treated plots and three control plots). Each plot was 8 meters (m) length and 1m in width (with a 1m interval between blocks), and 50 plants were planted in two rows in each plot. Plot 1, Plot 2 and Plot 3 were used to refer to three repetitions. To ensure the direct contact of the seedling roots with *R. irregularis* CD1, we added a suspension of approximately 1,000 spores near each seed, 3 cm under the surface of the soil. The matched controls were given the equivalent volume of blank M- suspension. (B) Disease centers in the field. In September, 2016 (at 120 dpi), we noticed three obvious disease centers, which were exactly distributed among the three control plots. Analogous design but different place was also used in 2015.

Table S1. Effects of AMF on the growth promotion of Lumian 1 at 55 and 72 dpi under field conditions.

Year	Plot	Mycorrhizal colonization	Plant height at 55 dpi (cm)	Stem diameter at 55 dpi (mm)	Plant height at 72 dpi (cm)	Stem diameter at 72 dpi (mm)
2015	Plot1	−	57.06±8.27 c	9.20±1.99 b	113.28±7.27 c	14.33±1.12 c
		+	60.77±5.66 ab	9.83±1.18 b	120.42±5.60 ab	15.45±0.79 ab
	Plot2	−	56.49±8.63 c	10.57±1.15 a	114.29±7.03 c	15.35±0.84 b
		+	60.53±5.08 ab	10.60±1.35 a	119.14±4.29 b	15.40±0.96 b
	Plot3	−	57.72±6.67 bc	9.86±2.37 b	115.26±5.02 c	15.08±1.17 b
		+	62.94±9.43 a	10.94±1.00 a	123.04±8.34 a	16.10±1.00 a
2016	Plot1	−	38.78±3.76 bc	9.21±1.81 c	111.73±4.63 c	14.10±1.12 c
		+	39.86±4.14 abc	9.4±1.64 bc	114.04±4.97 bc	14.92±0.71 b
	Plot2	−	38.22±3.40 c	9.18±1.67 c	113.73±4.08 c	14.85±1.17 b
		+	41.64±2.98 ab	10.09±1.71 ab	117.42±5.60 ab	15.53±1.12 a
	Plot3	−	38.74±4.33 bc	9.38±1.69 bc	112.26±5.02 bc	14.87±1.16 b
		+	42.34±4.91 a	10.25±1.54 bc	119.08±6.56 a	15.87±1.00 a

Mean values within columns of same year followed by different letters are significantly different at $P<0.05$. Error bars represent ±SD.

Table S2. qRT-PCR Primers used in this study.

Gene name		Primer sequences
Gh_A02G0202	Forward	5′-TCAATGCGGAACAAGAGAAG-3′
	Reverse	5′-CCGCAGAGTGCAATAAGTGT-3′
Gh_A02G0203	Forward	5′-CTTGCCATCCCTTACCAGCA-3′
	Reverse	5′-CCCTTTTTGTCCTTGATTTTGGC-3′
Gh_D02G0263	Forward	5′-TGCTGCACAAAGCACACATC-3′
	Reverse	5′-GCTCTGCTGGTGCCCATTAT-3′
Gh_D10G1372	Forward	5′-ATCGCTTATCGCACTTTGTG-3′
	Reverse	5′-ATTGAATTGTGAACCTGCCA-3′
GhUBQ7	Forward	5′-GAAGGCATTCCACCTGACCAAC-3′
	Reverse	5′-CAAAACTCCAAAATCATACCCAAAG-3′
V. dahliae	Forward	5′-AAAGTTTTAATGGTTCGCTAAGA-3′
	Reverse	5′-CTTGGTCATTTAGAGGAAGTAA-3′
GhHSR203J	Forward	5′-GTGATAGCTCAGGAGGGAACAT-3′
	Reverse	5′-CTAACTCGGACTTGCTTCGTTG-3′
GhHIN1-F	Forward	5′-GCTGATGAGACATCGGAGTTTA-3′
	Reverse	5′-CTACCATTCCCAGTGTTCAAAG-3′
GhNPR1-F	Forward	5′-TCAGTTTAGACAAGCCCGAGAA-3′
	Reverse	5′-CGTATGACCCTCTTTCAGTAGCA-3′
GhPR1	Forward	5′-AGACTACCTCAACGCTCACAACAC-3′
	Reverse	5′-CAACCCACATATTTACAGCATCG-3′
GhPR3	Forward	5′-CGTGGTGCCTTGCCTATCTATTG-3′
	Reverse	5′-GCGTCGTTCTTGGTCGGTTTC-3′
GhPR4	Forward	5′-CGGGGTGTTCCTTGATGCTC-3′
	Reverse	5′-TTACGCTCCACCGTCCTCTTC-3′
GhPR5	Forward	5′-GCCAGGGATTCTATCAAACGC-3′
	Reverse	5′-ATCCTCGGAGCAATGGGTTC-3′
GhPR9	Forward	5′-CCCAACTGTGAGCGAGGAAT-3′
	Reverse	5′-GGCGACGACACCAGCAAG-3′
GhPR10	Forward	5′-ATGACTCTGCCTCCCCTGACG-3′
	Reverse	5′-CCCGCCTTCAATCTAACTGTATCTG-3′
GhPAL5	Forward	5′-CCAAGTGGCTAAAAGAGTCCTAACA-3′
	Reverse	5′-GGTCGTCGGCGTAGGCATAGA-3′
GhC4H1	Forward	5′-CCGAACCCGACACCCATAAGC-3′
	Reverse	5′-GCAGGGATGTCATACCCACCAAG-3′
GhHCT1	Forward	5′-CCTGGTTACTTTGGGAATGTGAT-3′
	Reverse	5′-CCTTAGATATTCATCATCCATCCGA-3′
Gh4CL1	Forward	5′-GTGTCTTGCCTTTATTCCACATTTAC-3′
	Reverse	5′-TTCTTAGCCAACAACACCACCAAC-3′
GhCAD1	Forward	5′-GACTTTGGTAGTTGTGGGGGC-3′
	Reverse	5′-GAGACGCAAGAAATGATGGATGT-3′
GhLOX1	Forward	5′-TGATGGACTGGAGATTTGGTTTG-3′
	Reverse	5′-GCTTGAAGTTCAGGGTCTTGTTG-3′
GhACO1	Forward	5′-CTCCCAAGCCCACAAAAGTT-3′
	Reverse	5′-ACAGAGTCCAGCCGTGATGC-3′
GhOPR3	Forward	5′-TGGAAGATGATTGTGGATGCTG-3′
	Reverse	5′-GAGATGGGTTTGTTTGTTGAGGA-3′
